# Dietary Protein From Different Sources Exerted a Great Impact on Lipid Metabolism and Mitochondrial Oxidative Phosphorylation in Rat Liver

**DOI:** 10.3389/fnut.2021.719144

**Published:** 2021-08-26

**Authors:** Xuebin Shi, Zixin Huang, Guanghong Zhou, Chunbao Li

**Affiliations:** ^1^Key Laboratory of Meat Processing and Quality Control MOE, Nanjing Agricultural University, Nanjing, China; ^2^Key Laboratory of Meat Processing MOA, Jiangsu Synergetic Innovation Center of Meat Processing and Quality Control, Nanjing Agricultural University, Nanjing, China

**Keywords:** dietary protein, proteome, liver biotransformation, inflammation, antioxidation

## Abstract

Associations between meat diets and human health have been widely considered. In this study, we focused on long-term effects of different sources of meat protein on liver metabolic enzymes. For 90 days, rats were fed with semisynthetic diets that differed only with protein source. Casein was used as a reference and isolated soybean, fish, chicken, pork, and beef proteins were compared. Changes in liver proteome were determined by isobaric tag for relative and absolute quantitation (iTRAQ) labeling and liquid chromatography electrospray ionization tandem mass spectrometry/mass spectrometry (LC–ESI–MS/MS). Fish and pork protein diets upregulated the gene expression involved in cholesterol synthesis and esterification, and pork protein diet also upregulated the gene expression of high-density lipoprotein receptor and low-density lipoprotein receptor. Chicken, pork, and beef protein diets upregulated the gene expression involved in cholesterol reverse transport and bile acid production, which increased the total cholesterol level in the fish protein diet group. Total cholesterol levels in liver were lower in the pork and beef protein diet groups. Triglyceride levels in liver were lower in chicken, pork, and beef protein diet groups. Peroxisomal proliferator-activated receptor-gamma coactivator-1 was upregulated by chicken, pork and beef protein diets, and promoted the degradation and metabolism of triglyceride, resulting in lower triglyceride in the three diet groups. Meat proteins at a recommended level could be more conducive to cholesterol degradation, triglyceride decomposition, and energy balance maintenance at a healthy level. The findings give a new insight into the associations between meat diet intake and human health.

## Introduction

Dietary protein provides the basic nitrogen source necessary for life, and participates in a series of physiological processes such as energy metabolism (1), hormone secretion (2), appetite control (3), and lipid metabolism (4). Descovich et al. (5) found that protein diets could regulate lipid synthesis and metabolism. Many studies have focused on effects of high protein and protein restriction in diets on growth performance; however, only few data are available on the diet effect on liver metabolism.

Previous studies have shown that diets may differentially regulate the gene expression of enzymes and transporters involved in lipid metabolism. For example, whey protein (6), soybean protein (7–9), and fish protein (10) have a great impact on lipid metabolism (11). We have investigated the effects of different dietary meat proteins on gene expression in rat liver by transcriptome method, and proposed the relationships of body weight, blood lipid, and amino acid levels with hepatic lipid metabolism, amino acid metabolism, and pancreatic islet signaling pathway (12). Long-term diet intake is a better reflection of the effects of different protein diets on growth performance and physiological responses (13). However, only few data are available on the effects of meat protein diets on lipid metabolism, and on its underlying mechanisms.

In this study, we explored the proteomic changes in rat liver with different protein diets, in particular to the signal pathways and their regulatory physiological functions. Molecular biological methods were used to verify the differential expression and reveal the potential mechanisms. Bioinformatics tools were used to predict upstream regulatory factors.

## Materials and Methods

### Diets and Animals

Animal diets were prepared according to the recommendation of the American Institute of Nutrition (AIN-93) to meet the nutritional requirements for growing rats. All the diets are balanced for energy, macronutrient, and nitrogen content, which are presented in Table 1.

**Table 1:** Composition and nutrients of the diets.

	**Casein**	**Soybean**	**Pork**	**Beef**	**Chicken**	**Fish**
**Ingredient composition (g/kg)**						
Protein powder^a^	200	200	190	191	192	193
Corn starch	397.5	397.5	397.5	397.5	397.5	397.5
Corn dextrin	132	132	132	132	132	132
Saccharose	100	100	100	100	100	100
Soybean oil	70	70	70	70	70	70
Cellulose	50	50	50	50	50	50
Me minerals^b^	35	31.2	30.3	30.2	27.8	27.9
Vitamins^c^	10	10	10	10	10	10
L-Cystine^d^	3	3	3	3	3	3
Choline bitartrate	2.5	2.5	2.5	2.5	2.5	2.5
Water	0	3.8	14.7	13.8	15.2	14.1
Total	1,000	1,000	1,000	1,000	1,000	1,000
**Nutritional content**						
Energy (kcal/kg)	4,130	4,130	4,130	4,130	4,130	4,130
Fats (g/kg)	70	70	70	70	70	70
Carbohydrate (g/kg)	696.9	696.9	696.9	696.9	696.9	696.9
Protein (g/kg)	178	178	178	178	178	178

a *According to the protein contents in protein powder from different sources, the amounts were balanced and adjusted to the same level*.

b *After determining the minerals in the different protein powders, some extra parts were added in the diets as supplements*.

c *The formulation of vitamins was as described by Reeves et al. (14)*.

d *The amino acid composition of the soybean and meat protein diets was not modified*.

Meat proteins were extracted from beef *longissimus dorsi* muscle, pork *longissimus dorsi* muscle, chicken *pectoralis major* muscle, and silver carpback muscle obtained from a local meat company (Sushi, Jiangsu, China) as described previously (15). The processes include fat-removing, freeze-drying, and powder preparation. The preparation steps of meat protein powder were as follows: the visible connective tissue was removed, and the muscles were ground and placed in plastic bags and cooked in a water bath at 72°C, with a central temperature of 70°C. The cooked samples were cooled and freeze-dried for 36 h. The freeze-dried meat samples were ground into powder, and fat and moisture were removed with dichloromethane and methanol (v/v = 2/1). The powders were passed through a 25-mesh sieve. The protein content in the powders was >90%.

Casein and soy protein were obtained from Jiangsu Teluofei Inc. (Nantong, China) and Linyi Shansong Biological Products Inc. (Linyi, China), respectively. Soy protein isoflavones were removed by alcohol extraction.

All animals were handled in accordance with the guidelines of the Ethical Committee of Experimental Animal Center of Nanjing Agricultural University. Sixty-six male Sprague–Dawley rats (4-week-old, 117 ± 10 g) were purchased from Zhejiang Experimental Animal Center (Hangzhou, China) and reared in a specific pathogen-free animal center. After 1-week acclimation to new environment by feeding with the nutritionally balanced semisynthetic diets (14), the rats were assigned randomly to six formulated diets with proteins isolated from pork, beef, chicken, fish, soy, and casein (*n* = 11 for each diet). The rats were fed using different sources of protein diets for 90 days. The animals were housed individually in plastic cages and given water and diets *ad libitum* in a temperature –(20 ± 0.5°C) and humidity –(60 ± 10%) controlled room with a 12-h light–dark cycle.

### Quantitative Proteomic Analysis

#### Sample Collection and Protein Preparation

After 90 days of feeding, the rats were fasted for 4 h and then killed by head dislocation. Liver samples were obtained, snap-frozen in liquid nitrogen, and stored at −80°C until analysis. Proteins were extracted from the liver samples as described previously (12, 15), with minor modifications. Briefly, 0.1 g liver samples were placed in a 1-ml protein lysis buffer (containing protease inhibitors) and homogenized intermittently in ice bath at 7,500 rpm. The samples were centrifuged at 4°C, 16,000 g for 1 h. The supernatant was collected and mixed with 5 × volume of chilled acetone containing 10% (v/v) trichloroacetic acid (TCA) and kept at −20°C for 4 h. Then, the samples were again centrifuged at 16,000 g at 4°C for 15 min, and the supernatant was discarded. The pellet was washed with chilled acetone three times, air-dried and dissolved in a urea buffer (8 M urea, 0.1 MHCl, pH8.5), sonicated, and then centrifuged. The supernatant was transferred to a new tube for quantification of protein concentration.

Then, 150 μg protein was mixed with a 400-μl urea buffer in an ultrafiltration tube and centrifuged, and the filtrate was discarded. Dithiothreitol (DTT, 10 mM) was added to the mixture, which was then incubated at 56°C for 1 h to reduce the disulfide bonds in proteins of the supernatant. Subsequently, 55 mM iodoacetamide (IAM) was added to block the cysteine and then the mixture was incubated for 1 h in the dark. The supernatant was mixed with a 100-μl buffer (from iTRAQ kit). After centrifugation at 30,000 *g* at 4°C for 20 min, the supernatant was discarded. Suspension and centrifugation were repeated twice to remove iodoacetamide (IAM). The protein was resuspended in a sample buffer and digested by trypsin (Promega, Madison, WI, United States) at a for protein to trypsin (v/v) ratio of 30:1 at 37°C for 16 h. The digest was centrifuged for 30 min, and the precipitation containing peptides was collected and dried by vacuum centrifugation.

#### ITRAQ Labeling and High pH Reversed Phase Fractionation

Peptides (20 μg) were labeled with an 8-plex isobaric tag for relative and absolute quantitation reagent (Applied Biosystems, Waltham, MA, Unites States) according to the protocol of the manufacturer. In brief, a unit of the iTRAQ reagent was reconstituted in 24 μl of isopropanol. Then, the iTRAQ reagent was mixed with the samples. Labeled peptides were incubated at room temperature for 2 h. Six labeled samples from each group of different protein sources were pooled for comparison and dried by vacuum centrifugation.

To reduce sample complexity, we performed high pH reversed phase fractionation (HPR-PF) chromatography for the fractionation of isobaric tag for relative and absolute quantitation-labeled peptides. First, the peptide mixtures were reconstituted in 100 μl of buffer A (98% acetonitrile, 2% H_2_O) and loaded onto an ACQUITY UPLC BEH C18 2.1 × 100 mm column (1.7 μm, Waters Corp., Milford, MA, United States). The peptides were eluted at a flow rate of 0.2 ml/min using a gradient of 97–3% buffer A, and 3–97% buffer B (2% acetonitrile, 98% H_2_O) for 60 min. Elution was monitored by measuring the absorbance at 214 nm, and fractions were collected every 1 min. The eluted peptides were pooled into 60 fractions. According to the differences in fraction polarity (fraction collection time), these fractions were merged into eight mixing samples to improve test efficiency, and then vacuum-dried.

#### Nano LC-MS/MS Conditions

Identification of the samples was performed as described previously (16), with minor modification. Each of the fractions was dissolved in 0.1% formate and then centrifuged at 14,000 g for 20 min. The supernatant containing 1.5 μg peptides was loaded onto the Acclaim PepMap100 C18 column (100 μm × 2 cm, 5 μm, 100 Å, Thermo Fisher Scientific, Waltham, MA, United States), and the peptides were eluted on an analytical column (Acclaim PepMap® RSLC, C18,75 μm × 10 cm, 3 μm, 100 Å, Thermo Fisher Scientific, Waltham, MA, United States) by a gradient of 97–3% buffer A (0.1%), and 3–97% buffer B (80% acetonitrile, 0.1% formate) at a flow rate of 0.3 nl/min for over 160 min.

Data-dependent tandem mass spectrometry (MS/MS) was performed using an LTQ-Orbitrap XL mass spectrometer (Thermo Fisher Scientific, Waltham, MA, United States), equipped with a nano-electrospray ion source. The electrospray voltage applied was 2.2 kV. Intact peptides were detected in the Orbitrap at a resolution of 60,000. For MS scans, the m/z scan range was 300–1,600 Da, and automatic gain control target for full MS was 1–6. The five most prominent ions were selected for MS/MS analysis if they exceeded a threshold of 5,000 counts and were at least doubly charged. The normalized collision energy for high collision dissociation (HCD) was set to a value of 40%, and the resulting fragments were detected with 7,500 resolution in the Orbitrap. Every ion selected for fragmentation was excluded for 60 s by dynamic exclusion.

#### iTRAQ Data Analysis

Raw data were analyzed with the Proteome Discoverer software (version: 1.4, Thermo Fisher Scientific, Waltham, MA, United States). Protein identification was performed using the Sequest HT search engine against the UniprotKB *Rattus Norvegicus* database. Searching parameters were set as follows: trypsin was chosen as the enzyme with allowance at most two missed cleavage; Gln → pyro-Glu (Q) @N-term, oxidation (M), deamidated as potential variable modifications, and carbamidomethyl (C), iTRAQ 8plex (N-term), and iTRAQ 8plex (K) as fixed modifications. A mass tolerance of 10 ppm was permitted for intact peptide mass and 0.02 Da for fragmented ions. A percolator algorithm was applied to estimate the false discovery rate based on q-value, and only peptides at the 99% confidence interval were counted as identified protein. For protein quantitation, a protein had to contain at least two unique peptides. When the average of |fold change (FC)| ≥ 2 in experimentally treated groups (fish: F; chicken: C; pork: P; beef: B) compared with the control group (soybean: S; casein: L), the protein was considered to be a differential abundance protein.

### Lipid Content Analysis

The liver samples (0.2 g) were homogenized intermittently in 1.8 ml ice-cold physiological saline at 7,500 rpm for 2 min. The homogenates were centrifuged at 4°C, 3,500 g for 15 min. The supernatants were collected, and protein concentration was quantified. Triacylglycerol and total cholesterol were extracted with isopropyl alcohol and analyzed with commercial kits according to the protocol of the manufacturer (Nanjing Jiancheng Bioengineering Institute, Nanjing, China).

### Reverse Transcription Polymerase Chain Reaction (RT-PCR)

Total ribonucleic acid was extracted from the liver samples using RNA Reagent Kit (No.9796, Takara, Tokyo, Japan) according to the instructions of the manufacturer. The purity and quantity of the total RNA were measured with a Nanodrop 2000 spectrophotometer at 260 and 280 nm. The RNA (500 ng) was reversely transcribed into first-strand cDNA using Prime Script RT Master Mix Kit (No. RR036A, Takara, Tokyo, Japan) according to the protocol of the manufacturer. The RT-PCR reactions were run using SYBR®Premix Ex Taq™ (No. RR420A, Takara, Tokyo, Japan) in QuantStudio™ 6 Flex Real-Time PCR System (Thermo Fisher Scientific, Waltham, MA, United States). Primers were designed according to the public database at the National Center for Biotechnology Information (NCBI) and were synthesized by Sangon Biotech Co., Ltd (Sangon, Shanghai, China). The primers used for RT-PCR are presented in Table 2. Amplification was performed in a total volume of 20 μl, containing 10 μl of SYBR Premix Ex Taq, 0.4 μl of each primer (10 μM), 0.4 μl of ROX Reference Dye II, 2 μl of cDNA, and 6.8 μl of sterilized doubled-distilled water. The RT-PCR program was set as follows: 95°C for 30 s, 40 cycles of 95°C for 5 s, 60°C for 34 s, and 95°C for 15 s, held at 60°C for 1 min. The amplification efficiency of all the primers ranged from 90 to 105%. Piece sampling for each rat was performed in triplicate. Relative mRNA levels were calculated using the 2^−ΔΔCt^ method. Glyceraldehyde-3-phosphate dehydrogenase (*Gapdh*) or18 S ribosomal RNA (*Rn18s*) was applied as reference gene to determine peroxisome proliferator-activated receptor alpha (*Ppara*), *Pparg*, sterol regulatory element-binding transcription factor 1 (*Srebf1*), *Srebf2*, 3-hydroxy-3-methylglutaryl-Coenzyme A reductase (*Hmgcr*), cytochrome P450 family 7 subfamily A member (*Cyp7a1*), *Cyp27a1*, lecithin-cholesterol acyltransferase (*Lcat*), low-density lipoprotein receptor (*Ldlr*), scavenger receptor class B type I (*Scarb1*), ATP-binding cassette subfamily A member 1 (*Abca1*), acetyl-Coenzyme A acetyltransferase 2 (*Acat2*), *Ppargc1a*, uncoupling protein 1 (*Ucp1*), *Ucp*2, cytochrome c oxidase subunit I (*Cox1*), *Cox2*, mitochondrially encoded educed form of nicotinamide-adenine dinucleotid dehydrogenase 5 (*mt-Nd5*), and mitochondrially encoded cytochrome B (*mt-Cytb*).

**Table 2:** Primer sequences of the target and reference genes.

**Gene**	**Primer**	**Sequence 5^′^-3^′^**	**Accession**	**Length**	**Location**
*Ppara*	Forward	GGCGAACTATTCGGCTAAAG	NM_013196.2	88	757
	Reverse	CAGTACTGGCATTTGTTCCG			
*Pparg*	Forward	TACCACGGTTGATTTCTCCA	NM_013124.3	137	281
	Reverse	CAGGCTCTACTTTGATCGCA			
*Srebf1*	Forward	CCAGGTGACCCGACTATTCT	NM_001276708.1	63	2331
	Reverse	GGCTGAGCGATACAGTTCAA			
*Srebf2*	Forward	CTCACTCTCTGGAAAGGCCG	NM_001033694.2	104	2903
	Reverse	CAGAAGTAGTGCCGCTGACA			
*Hmgcr*	Forward	GAGACTTCGGGCAGAGCTAC	NM_013134.2	240	1272
	Reverse	GTGCGTCTCCATGAGGGTTT			
*Cyp7a1*	Forward	AGAGAATCATTAGCCGTGCCA	NM_012942.2	286	2577
	Reverse	AGGGAGACATTTGAGTGAGCG			
*Cyp27a1*	Forward	GAGTGCATCAGGGGATCAGG	NM_178847.3	144	876
	Reverse	GATCTGATGAAGGTGGCGGT			
*Lcat*	Forward	ACTCAGTAACCACACACGGC	NM_017024.2	110	130
	Reverse	TCTTTCGGTAGCACAGCCAG			
*Ldlr*	Forward	GTCCTCCCAAGTCCAAGGTG	NM_175762.3	155	2512
	Reverse	TAATGTTCCTCAGCCGCCAG			
*Scarb1*	Forward	TGATGCCCCAGGTTCTTCAC	NM_031541.2	147	1509
	Reverse	CCTTATCCTGCGAGCCCTTT			
*Abca1*	Forward	ACCCATACTCTCGCAG	NM_178095.3	216	3513
	Reverse	CCACATCTTTCTTGACC			
*Acat2*	Forward	GGGTGCAACATTTCCGAACC	NM_153728.3	185	625
	Reverse	CGTGGACAGGAACATGGGAA			
*Ppargc1a*	Forward	CCACTACAGACACCGC	NM_031347.1	165	1853
	Reverse	CTTTCAGACTCCCGCT			
*Ucp1*	Forward	TATCATCACCTTCCCG	NM_001106591.1	67	1346
	Reverse	TGCCACACCTCCAGTC			
*Ucp2*	Forward	TTCTATGGGAAATCAAGGGG	NM_019354.3	125	159
	Reverse	CGGAGTCGGGAGGGTG			
*Cox1*	Forward	AGTATTAGCAGCAGGTATCAC	MW209726.1	118	5924
	Reverse	GCCGAAGAATCAGAATAGGT			
*Cox3*	Forward	CCGTGAAGGAACATACCAA	MW209726.1	235	8786
	Reverse	TGATGCTAAGAGGACTGATG			
*mt-Nd5*	Forward	CTCATCAGTAAGCCATATAGC	MW209726.1	146	11025
	Reverse	TTCGTTCGTAGTTGGTGTT			
*mt-Cytb*	Forward	ACTTCGGTTCTCTACTAGGA	MW023797.1	196	95
	Reverse	TGGAGGAATAGGCAGATGA			
*Rn18s*	Forward	GGGGAGTATGGTTGCAAAGC	NR_046237.2	191	1169
	Reverse	CGCTCCACCAACTAAGAACG			

### Immunoblotting for Glucocorticoid Receptor (GR)

Western blot techniques were used to determine protein level. Liver tissues (100 mg) were treated with a lysis buffer (Nanjing Jiancheng Bioengineering Institute, Nanjing, China). Whole protein was quantified with an enhanced bicinchoninic acid assay (BCA) protein assay kit. The samples were mixed with a loading buffer and denatured by boiling for 5 min. Protein (40 μg) was loaded onto a 10% polyacrylamide gel electrophoresis (SDS-PAGE). Electrophoresis was performed at 80 V for 1.5 h at 4°C. Then, the proteins were blotted by electrodiffusion for 1.5 h at 90 V on nitrocellulose membranes. Blotted membranes were blocked with 5% skim milk in Tris-buffered saline containing 0.1% Tween 20 (TBST) for 2 h and then incubated with a rabbit polyclonal GR antibody (ab109022, Abcam, Cambridge, United Kingdom) for 12 h at 4°C. After being washed six times in tris-buffered saline and Tween 20 (TBST), the blotted membranes were incubated with goat anti-rabbit IgG (immunoglobulin G, BS13278, Bioworld, St. Louis Park, MN, USA) for 2 h. Target protein was detected with Image Quant LAS 4000 (GE Healthcare Life Sciences, Chicago, IL, United States). The intensity of the target protein was normalized against glyceraldehyde-3-phosphate dehydrogenase (*Gapdh*, MB001, Bioworld).

### Bioinformatics and Statistical Analysis

In this study, a protein expression matrix was generated with DataMerge2 and normalized to obtain differential abundance proteins by *t*-test. A multi-omics data analysis tool, OmicsBean (http://www.omicsbean.cn), which integrated Gene Ontology (GO) enrichment, was used, and Kyoto Encyclopedia of Genes and Genomes (KEGG) pathway analysis performed to analyze differentially expressed proteins. Means were compared by Duncan's multiple comparison under the SAS system (version 9.2). The significance level was set if *p* < 0.05 for all statistical analyses.

## Results

### Effect of Different Proteins Diets on Body Weight in Rats

There was no significant difference in the initial body weight of rats in the six groups (*p* > 0.05, Table 3, as shown in the previous article (17)). From weeks 2–5, the average body weight of rats fed with fish protein was greater than that of rats fed with soybean protein (*p* < 0.05). From weeks 6–11, the average body weight of rats fed with fish protein diet was significantly higher than that of rats fed with soybean, pork, and beef protein diets (*p* < 0.05). From weeks 12–13, the average body weights of rats fed with casein and fish protein diets were significantly higher than that of rats fed with soybean, pork, and beef protein diets (*p* < 0.05). The average daily weight gain of rats in the beef protein group was significantly lower than that in the casein and fish protein groups (*p* < 0.05).

**Table 3:** Effect of different proteins diets on body weight in rats (*n* = 10).

**Weeks**	**Casein (g)**	**Soybean (g)**	**Pork (g)**	**Beef (g)**	**Chicken (g)**	**Fish (g)**
0	165 ± 15	167 ± 12	167 ± 14	174 ± 14	167 ± 11	171 ± 10
1	262 ± 10	243 ± 15	263 ± 23	266 ± 18	261 ± 14	268 ± 12
2	333 ± 16^ab^	297 ± 19^b^	315 ± 17^ab^	330 ± 27^ab^	325 ± 7^ab^	342 ± 12^a^
3	401 ± 35^ab^	365 ± 18^b^	387 ± 16^ab^	398 ± 36^ab^	392 ± 24^ab^	419 ± 16^a^
4	438 ± 37^ab^	406 ± 18^b^	420 ± 21^ab^	431 ± 45^ab^	430 ± 26^ab^	454 ± 18^a^
5	482 ± 47^a^	443 ± 25^b^	463 ± 21^a^	470 ± 48^a^	470 ± 35^a^	499 ± 28^a^
6	531 ± 54^ab^	493 ± 32^b^	508 ± 26^b^	509 ± 53^ab^	522 ± 41^ab^	550 ± 31^a^
7	572 ± 61^ab^	518 ± 48^c^	536 ± 20^bc^	532 ± 50^bc^	556 ± 47^abc^	586 ± 34^a^
8	598 ± 72^ab^	528 ± 72^d^	566 ± 26^bcd^	549 ± 48^cd^	581 ± 56^abc^	610 ± 36^a^
9	616 ± 75^ab^	554 ± 67^d^	586 ± 25^bcd^	564 ± 47^cd^	598 ± 59^abc^	629 ± 36^a^
10	638 ± 83^ab^	571 ± 65^c^	602 ± 36^bc^	576 ± 52^c^	609 ± 60^abc^	638 ± 37^a^
11	656 ± 87^ab^	587 ± 66^c^	621 ± 43^bc^	590 ± 53^c^	623 ± 60^abc^	663 ± 41^a^
12	673 ± 94^a^	615 ± 59^b^	630 ± 43^b^	600 ± 61^b^	639 ± 74^ab^	676 ± 42^a^
13	687 ± 99^a^	630 ± 61^b^	644 ± 41^b^	610 ± 70^b^	650 ± 76^ab^	686 ± 43^a^
ADG(g/d)	5.82^a^	5.19^ab^	5.26^ab^	4.83^b^	5.38^ab^	5.72^a^

*ADG means average daily weight gain; the different superscript lowercase letters in the same line show significant difference (p < 0.05)*.

### The General Proteome Profile in Rat Liver Varied With Dietary Proteins

**Figure 1:**
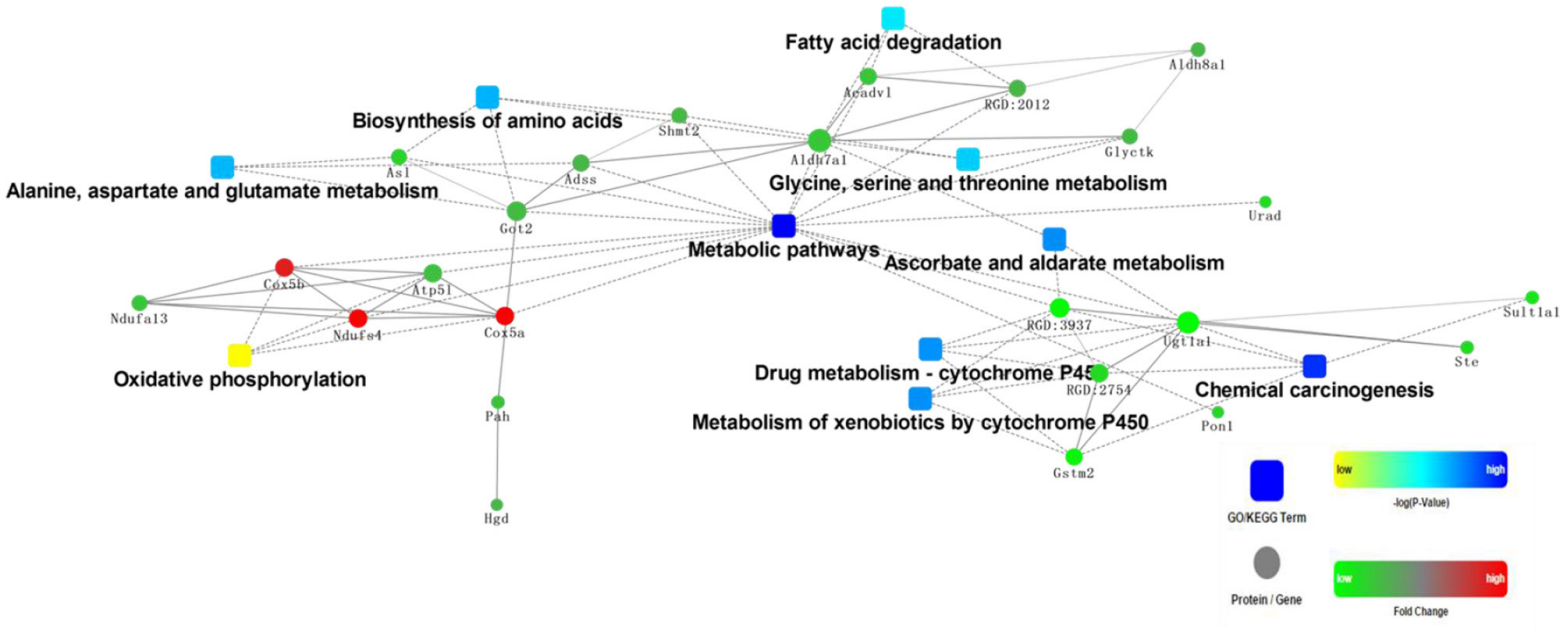
Protein-protein interaction analysis of rat liver differentially expressed protein in chicken: casein. Squares are pathways, and dots are proteins. Yellow squares and green dots represent downregulation, while blue squares and red dots represent upregulation.

Generally, dietary proteins have a great impact on proteome profiles in rat liver. For example, compared with the casein diet group, intake of chicken protein diet regulated proteins involved in various metabolic processes and oxidative phosphorylation (Figure 1). Of these proteins, organic nitrogen metabolic pathways were significantly altered, such as valine, leucine, and isoleucine degradation, β-alanine metabolism, histidine metabolism, and biosynthesis of amino acids. Carbon metabolism and fatty acid degradation were also significantly regulated.

### Dietary Protein Regulated Energy Metabolism-Related Proteins in Rat Liver

**Figure 2:**
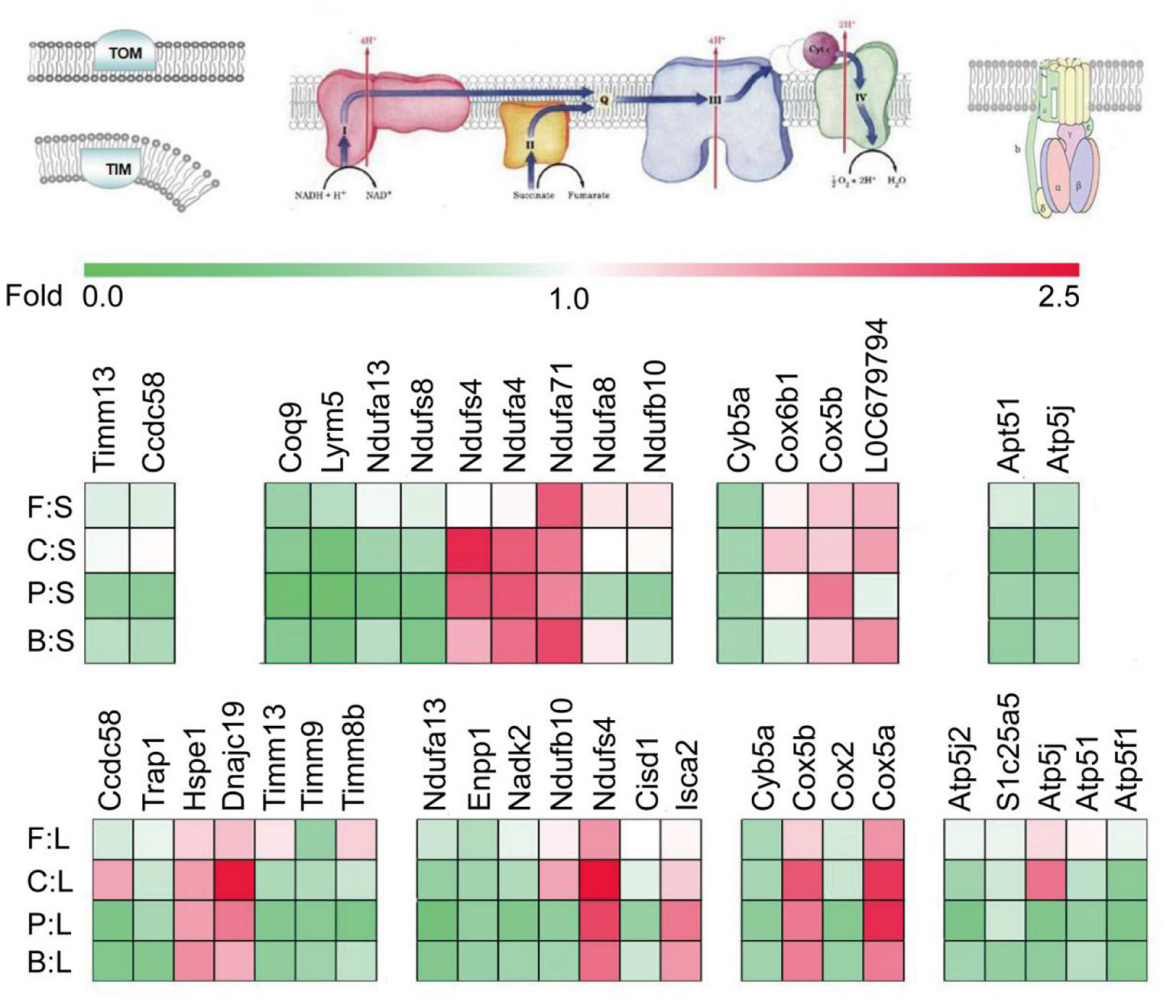
Effect of dietary casein, soybean, and meat proteins on rat liver mitochondrial respiratory chain-related protein expression. The colors of squares indicate the direction of changes in proteins, with red for up-regulation and green for down-regulation. F:S, fish protein vs. soybean protein; C:S, chicken protein vs. soybean protein; P:S, pork protein vs. soybean protein; B:S, beef protein vs. soybean protein; F:L, fish protein vs. casein; C:L, chicken protein vs. casein; P:L, pork protein vs. casein; B:L, beef protein vs. casein.

As shown in Figure 2, significant differences are found in liver mitochondrial protein transport, respiratory chain, and ATP synthesis among the diet groups. The levels of translocase of inner mitochondrial membrane (TIMM13, TIMM8B, and TIMM9) and chaperones, such as coiled-coil domain-containing 58 and TNF receptor-associated protein 1(CCDC58, TRAP1), were lower in the chicken, pork, beef, and fish protein diet groups. To a certain extent, the chicken, pork and beef protein diet groups showed low mitochondrial activity. However, compared with casein, the intake of chicken protein diet upregulated several heat shock proteins and their subclasses (HSPE1, DNAJC19) involved in mitochondrial protein transport, indicating a diet-induced selective transport of protein in mitochondria. The intake of pork, beef, and chicken protein diets downregulated ATP synthase subunits (ATP5F1, ATP5J, ATP5J2, and ATP5L) and ADP/ATP translocase like solute carrier family 25 member 5 (SLC25A5), indicating that meat protein diets could reduce the level of energy production in liver. The oxidation of mitochondrial substrate is coupled with the energy metabolism of ATP production by ADP phosphorylation. The ATP synthase was lower in the meat protein diet groups; thus, we speculated that in the absence of an uncoupling mechanism, there could be significant differences in the oxidation of a mitochondrial substrate and energy supply among the diet groups. In addition, proteins involved in dehydrogenation, such as ectonucleotide pyrophosphatase/phosphodiesterase 1 and NAD kinase 2 (ENPP1, NADK2); respiratory chain-associated proteins such as NADH: ubiquinone oxidoreductase subunit (NDUFA13, NDUFB10); electron transport-chain associated proteins such as CDGSH; and iron sulfur domain-containing proteins (CISD1, CYB5A, and COX2) were downregulated in all the meat protein diet groups. However, some proteins, such as Cox5a and Cox5b, and iron sulfur cluster-related proteins (ISCA2, NDUFS4) were highly expressed in the chicken protein diet group.

### Dietary Proteins Regulated on Gene Expression Involved in Cholesterol Metabolism in Rat Liver

**Figure 3:**
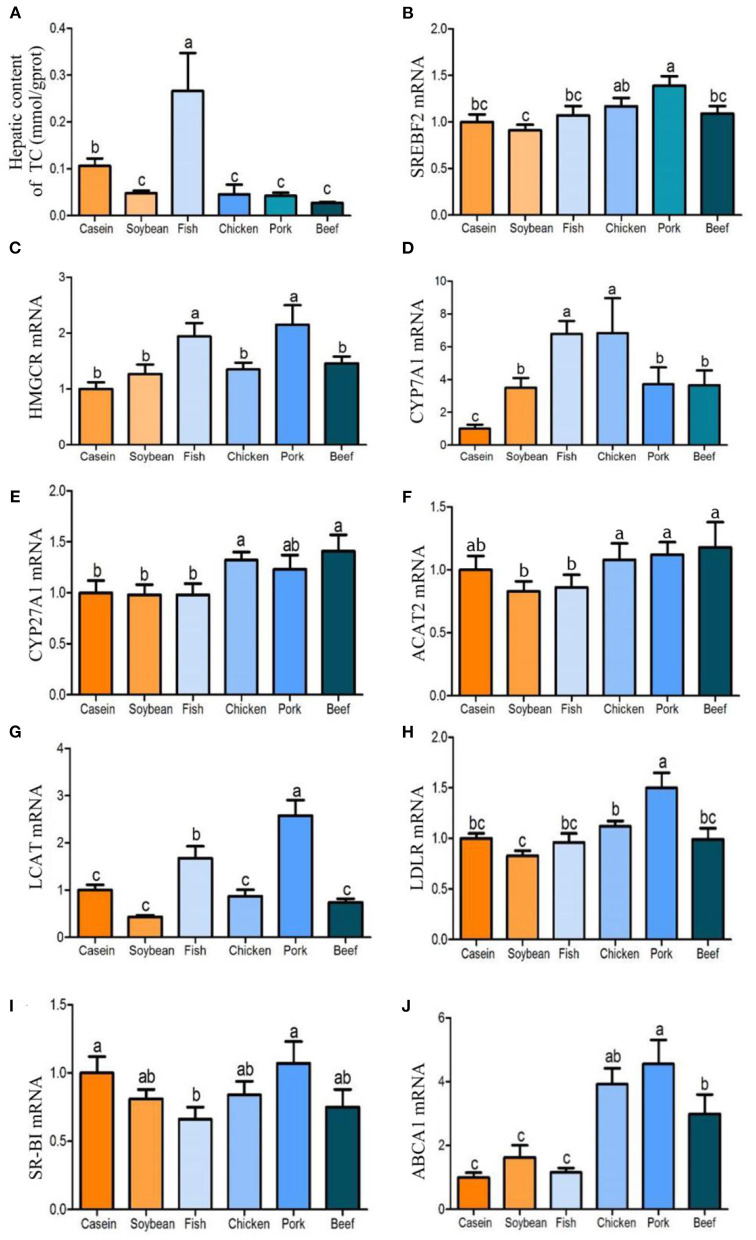
Effects of dietary casein, soy and meat proteins on liver total cholesterol and cholesterol metabolism related genes. **(A)** Total cholesterol in the liver of rats fed different protein diets. **(B–J)** qPCR analysis of cholesterol metabolism related genes. Values are presented as the means ± SD. Groups were compared by one-way ANOVA followed by Duncan's multiple range tests. Letters a–c represent significant differences between diet groups (*p* < 0.05).

After 90 days of feeding, the total cholesterol content in liver differed with the dietary protein source. The total cholesterol content in the liver of rats fed with fish protein diet was significantly higher than that of the other diet groups (Figure 3A). The total cholesterol contents in the liver of the chicken, pork, and beef protein diet groups were significantly lower than those of the casein and fish protein groups, but had no significant difference with that of the soybean protein group. In addition, the triglyceride contents of the chicken, pork, and beef protein groups were significantly lower than those of the soybean and casein protein groups. The triglyceride content in the fish protein group did not differ from those of the casein and soybean protein diet groups, but it was higher than those of pork and beef protein diet groups. Compared with the soybean and casein protein diet groups, *Srebf2* mRNA level in liver was highest in rats fed pork protein diet, which was also higher than those of the fish and beef protein diet groups, and the chicken protein diet group was significantly higher than that of the soybean protein diet group (Figure 3B). *Hmgcr* was the rate-limiting enzyme of cholesterol synthesis. The levels of *Hmgcr* in the fish and pork protein diet groups were significantly higher than those in the casein, soybean, chicken, and beef protein diet groups (Figure 3C). The expression of the *Cyp7a1* gene was significantly higher in the fish and chicken protein groups than in the casein, soybean, pork, and beef protein groups. The mRNA levels of the *Cyp7a1* gene in the pork and beef protein diet groups were significantly higher than that in the casein diet group, but had no significant difference with that in the soybean protein group (Figure 3D). The mRNA levels of the *Cyp27a1* gene in the chicken and beef protein diet groups were significantly higher than those in the casein, soybean, and fish protein diet groups (Figure 3E). The *Acat2* gene, which could catalyze free cholesterol esterification, did not differ with dietary protein source (Figure 3F). However, *Lcat*, which catalyzes lecithin and cholesterol esterification, was highly expressed in the pork and fish protein groups. This indicates that the levels of HDL cholesterol esterification in the blood of rats fed with pork and fish proteins were high, and that cholesterol was transported reversely into the liver, which was consistent with the high cholesterol content in the liver of the fish protein diet group (Figure 3G). *Abca1*, which catalyzes cholesterol reverse transport, was highly expressed at mRNA level in the chicken, pork, and beef protein groups, but there was no significant difference among the fish, casein, and soybean protein groups (Figure 3J). The mRNA levels of *Ldlr* and *Scarb1* in the chicken, pork, and beef protein groups showed similar changes to *Abca1*, but the mRNA level of the *Ldlr* gene was higher in the pork protein diet group than in other the diet groups, and the mRNA level of *Scarb1* was higher in the pork protein and casein diet groups than in the fish protein diet group (Figures 3H,I).

### Dietary Proteins Changed Gene Expression Involved in Lipid and Energy Metabolism

**Figure 4:**
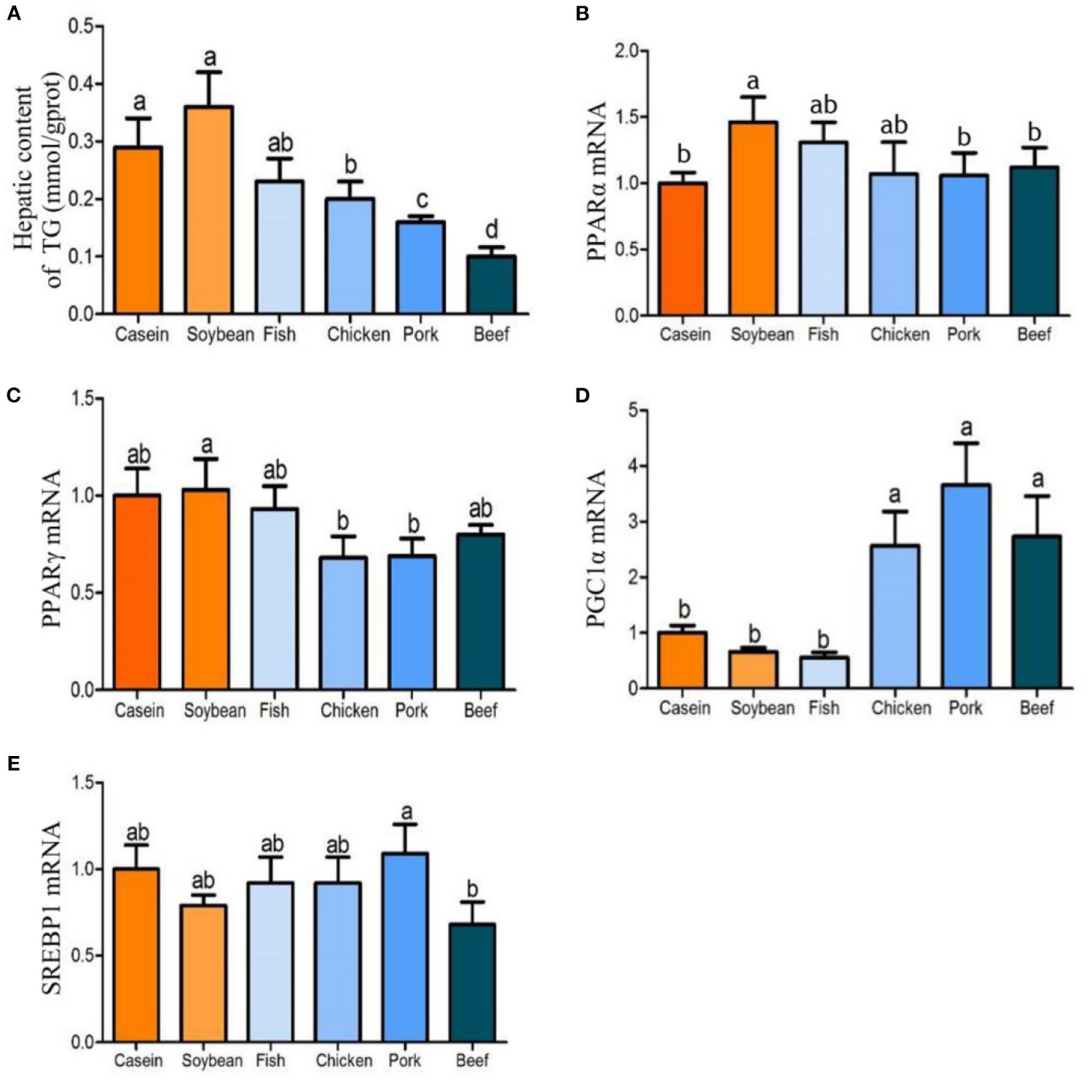
Effect of dietary casein, soy and meat proteins on rat liver lipid metabolic regulation factor related genes. **(A)** Triglyceride in the liver of rats fed different protein diets. **(B–E)** qPCR analysis of lipid metabolism related genes. Values are presented as the means ± SD. Groups were compared by one-way ANOVA followed by Duncan's multiple range tests. Letters a–c represent significant differences between diet groups (*p* < 0.05).

**Figure 5:**
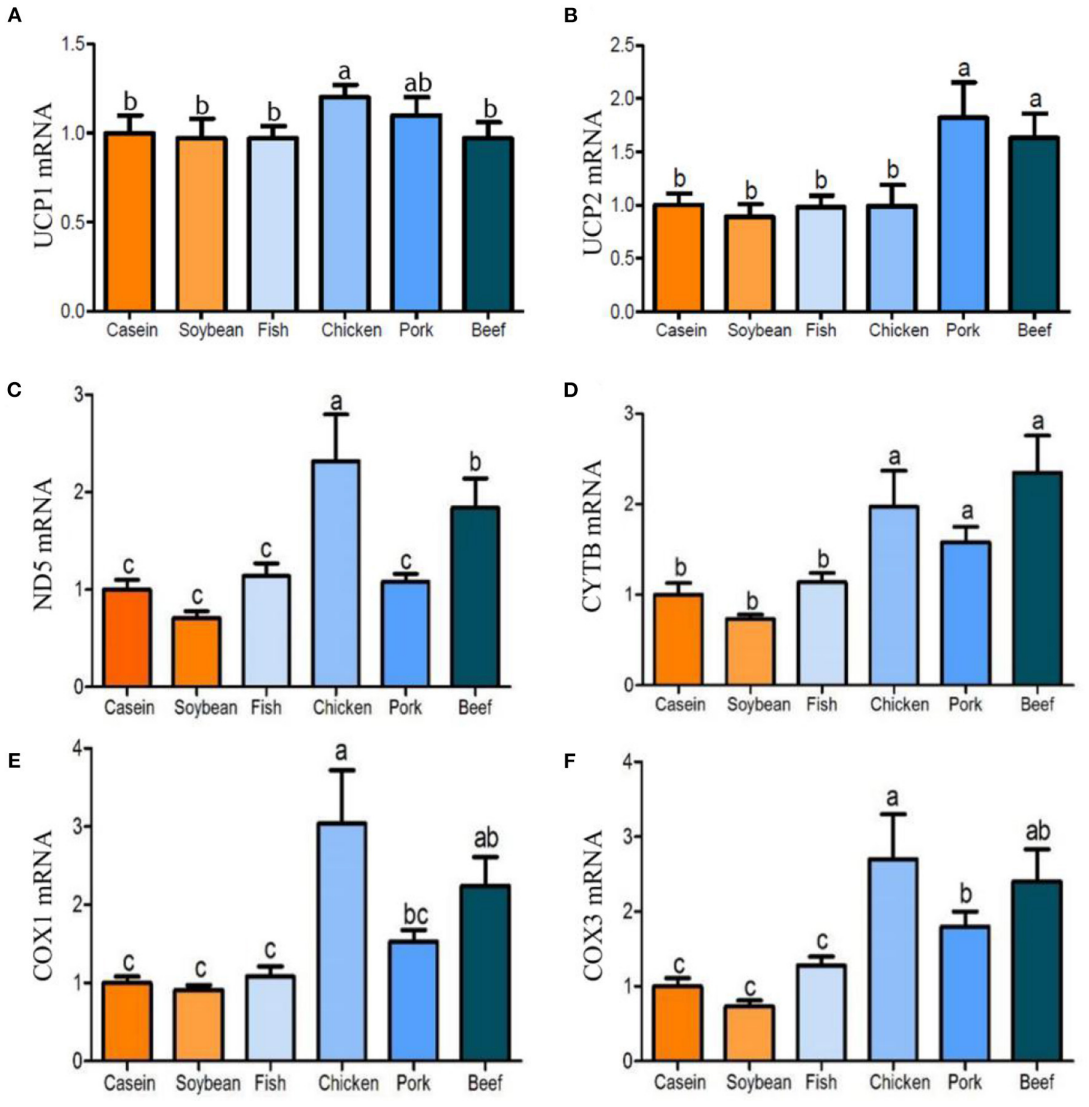
**(A–F)** Effect of dietary casein, soy and meat proteins on rat liver mitochondrial uncoupling and respiratory chain related genes. Values are presented as the means ± SD. Groups were compared by one-way ANOVA followed by Duncan's multiple range tests. Letters a–c represent significant differences between diet groups (*p* < 0.05).

After 90 days of feeding, the triglyceride contents in the liver of the chicken, pork, and beef protein diet groups decreased gradually, and were significantly lower than that in the soybean and casein diet groups (Figure 4A). There was no significant difference in the triglyceride contents among the casein, fish, and soybean protein groups, but higher than those of the pork and beef protein diet groups. No significant difference was observed in *Ppara*m RNA among all the diet groups (Figure 4B). The mRNA levels of *Pparg* were lower in the chicken and pork protein diet groups than in the soybean protein diet group (Figure 4C). This is confirmed by the previous study (12) in which the weight of epididymal fat and liver was lower in rats fed with pork and beef proteins. In addition, the mRNA levels of *Ppargc1a* were higher in the chicken, pork, and beef protein diet groups than those in the casein, soybean, and fish protein diet groups (Figure 4D). The *Srebp1* mRNA levels were different between the pork and beef protein diet groups (Figure 4E). The *Ucp1* mRNA level showed a change similar to that of *Ppargc1a* (Figure 5A). The *Ucp2*mRNA levels were also higher in the pork and beef protein diet groups than those in the casein, soybean, fish, and chicken protein groups (Figure 5B). The mRNA levels of *mt-Nd5, mt-Cytb, Cox1*, and *Cox3* were higher in the chicken, pork, and beef protein diet groups than those in the casein, soybean, and fish protein diet groups (Figures 5C–F).

### Dietary Proteins Affected the Gene Expression of Glucocorticoid Receptor in Rat Liver

**Figure 6:**
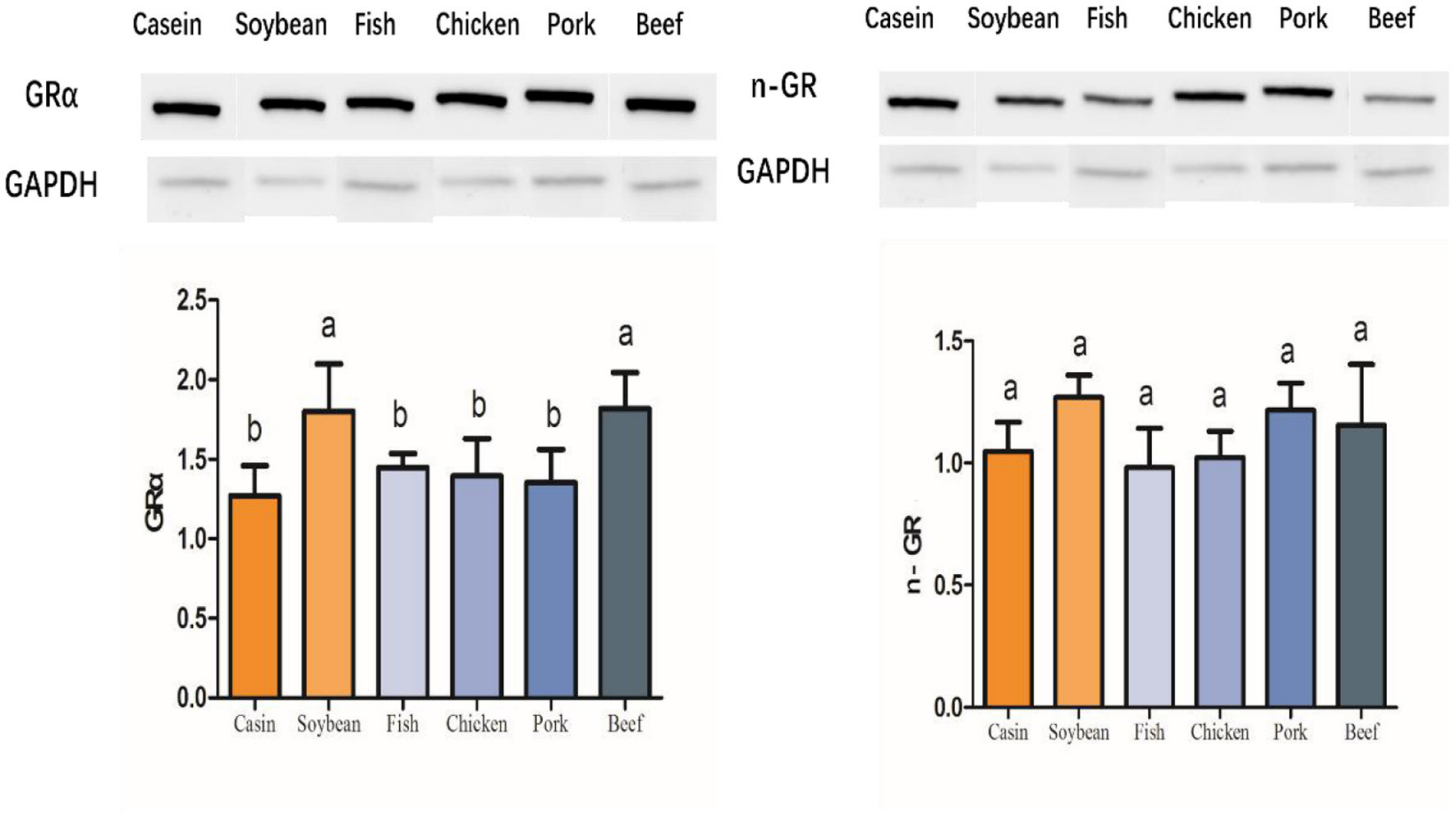
Western blot profiles of liver GRα and n-GR in the rats fed with different dietary proteins. The columns reflect the means and standard deviations of values in gray (*n* = 3). The groups were compared by one-way ANOVA followed by Duncan's multiple range tests. Letters a–c represent significant differences among the diet groups (*p* < 0.05).

Glucocorticoid is a kind of steroid hormone secreted by the adrenal cortex. It can regulate the biosynthesis and metabolism of sugars, fats, and proteins. It also has an anti-inflammatory effect. Glucocorticoid plays a role in regulating glucose and lipid metabolism by binding to glucocorticoid receptor (GR) (18). A genome-wide analysis showed that there is a GR binding site in the promoter of fatty acid synthesis gene in rats and other rodents, and that GR has some impact on the promoter activity of these genes (19). A proteomic study indicated that meat protein diets induced a differential 43kD subunit in rat liver, which was verified by Western blotting to be a part of the intact molecule as a glucocorticoid receptor (GRα) with a molecular weight of 87 kD (Figure 6). The relative abundance of this protein was higher in the soybean and beef protein diet groups than in the other groups. However, the low-weight molecule, that is, n-GR, did not differ among all the diet groups.

## Discussion

In previous studies (12, 16, 20), serum glucose, triglyceride, cholesterol, and other indicators were significantly lower in the beef protein diet group than in the casein, soybean, and other meat protein groups, while the serum triglyceride content was lower in the fish protein diet group than in the casein diet group, and the cholesterol levels were different between the chicken and beef protein diet groups. Diets may change the contents of glucose and free fatty acid in serum, and regulate the gene expression involved in glucose and fatty acid metabolism (21). The liver maintains the balance between glycogen storage and gluconeogenesis by sensing the blood glucose content in the portal vein (22). The degradation of dietary protein into amino acids establishes a relationship with glucose and lipid metabolism through the tricarboxylic acid cycle, and ultimately determines the metabolic pathway of intermediates in the branch point of fatty acid metabolism according to the demand of the body (23). At the same time, intermediates also regulate the expression of proteins involved in lipid metabolism.

### Effects of the Four Meat Protein Diets on Cholesterol Metabolism in Rat Liver

Cholesterol is widely distributed in various tissues of the body and is the main component of cell membrane. Majority of cholesterol is stored in the liver (24), which is also the main place for cholesterol synthesis, transport and regulation. As an important organ of fatty acid metabolism, liver consumes about half of non-esterified fatty acids in the serum (25). In this study, genes related to liver cholesterol synthesis (*Hmgcr*) and esterification (*Lcat*) were significantly higher in the fish and pork protein diet groups, while genes involved in cholesterol reverse transport (*Abca1*) and bile acid production (*Cyp27a1*) were significantly higher in the chicken, pork, and beef protein diet groups, resulting in higher hepatic cholesterol in rats fed with fish protein diet than in rats fed with chicken, pork, and beef protein diets. The expression of the low-density lipoprotein receptor (*Ldlr*) gene in the pork protein diet group was upregulated by cholesterol in a negative feedback way, which induces the liver to ingest cholesterol from LDL. On the contrary, the chicken and beef protein diet groups did not have such a phenomenon. Similarly, the high expression of the *Scarb1* gene in the pork protein diet group could promote the absorption of cholesterol in the liver and reduce cholesterol level through relatively high bile acid synthesis.

### Effects of the Four Meat Protein Diets on Lipid Metabolism in Rat Liver

Previous studies have shown that dietary proteins from different sources such as cattle, turkey, and pigs (26) can change lipid metabolism in the liver. Schwarz et al. (27) reported that dietary protein can change the gene expression of lipid metabolism and improve lipid accumulation in the liver. Ronis et al. (28) further studied the role of transcription factors in the regulation of lipid metabolism, and believed that soybean protein could upregulate transcription factors, such as *Ppara, Pparg, Lxra*, and downregulate the *Srebp1c* regulatory gene to improve lipid metabolism. Song et al. (12) compared the short-term effects of different meat protein diets on lipid metabolism in rat liver using proteomic and transcriptomic methods, and they found that chicken and fish proteins significantly inhibited fatty acid oxidation and the *Ppar* pathway. Moreover, fish and pork protein diets inhibited TCA cycle, oxidative phosphorylation, and electron transport chain. Song et al. (12) also found that meat protein diets downregulated *Ppars, Srebf1, Srebf2*, and *Scap*, which are involved in lipid and cholesterol metabolism. Srebf1, Srebf2, and *Scap* were considered to be involved in the differential regulation of lipid and cholesterol anabolism in the soybean and fish protein diet groups. In this study, the *Srebf1* gene was downregulated by the beef protein diet, while *Ppargc1a*, an important transcription coactivator, was upregulated by the chicken, pork and beef protein diets. It could promote the degradation of triglycerides, causing low levels of triglyceride in the chicken, pork, and beef protein diet groups.

### Effects of the Four Meat Protein Diets on Energy Metabolism in Rat Liver

A protein diet affects energy metabolism through the transformation of amino acid carbon chains (29). Glutamine is the most abundant amino acid in blood. It can be produced by glutaminase deamination, and then converted into α-ketoglutarate by glutamate dehydrogenase. By this way, glutamine enters the tricarboxylic acid cycle and plays an important role in maintaining the ATP level (30). Glutamate contents in meat proteins were significantly lower in the casein and soybean protein groups, but there was no significant difference in serum among the diet groups. This could be because the glutamic acid from casein and soybean protein directly participated in the oxidation, or indirectly through tricarboxylic acid cycle. Meat protein diets changed the expression of respiratory chain-related proteins in rat liver, and the ATP synthetase protein was significantly low. ATP is the main energy donor of anabolism, and also an important factor for regulating signaling pathways such as the mTOR pathway. The hydrolysis of ATP by vacuole h^+^ adenotriphosphatase (V-ATPase) is necessary for the amino acid regulation of V-ATPase-regulator and, thus, promotion of mTORC1 to lysosome and activate it (31). ATP synthesis is a part of the mTOR signaling pathway, and this process could be regulated by dietary amino acid composition and abundance.

In this study, a variety of genes involved in the electron transfer chain were highly expressed in the chicken, pork, and beef protein groups, but there were still differences among the meat protein diet groups. ATP production was significantly lower, indicating that there was an uncoupling phenomenon between substrate oxidation electron transfer and ATP synthesis. Takahashi et al. (32) showed that soybean protein significantly increased the mRNA level of *Ucp1* in brown adipose tissue of rats, and that *Ucp2, Ucp3*, and *Pparg2* tended to increase. In this study, *Ppargc1a*, which regulates adaptive heat production, was highly expressed in the chicken, pork, and beef protein diet groups. Similarly, *Ucp2* was also highly expressed in the pork and beef protein groups, indicating that pork and beef protein diets regulated energy metabolism, increased suitable heat production, and decreased ATP synthesis. This is consistent with the traditional idea of eating beef and mutton in winter to improve cold resistance. Studies have shown that glucocorticoid (GC) can inhibit the expression of *Ucp1* and *Ucp2* in brown adipose tissues and skeletal muscles of rats (33). In this study, the *Ucp2* mRNA level in rat liver was regulated by the dietary protein sources, and the glucocorticoid receptor showed different tendencies with diets. This indicates that dietary proteins may change energy metabolism and adaptive heat production through the *GC-GR-Ucp2* pathway.

## Conclusions

In this study, we investigated the relationships of dietary protein with cholesterol metabolism, lipid metabolism, and energy synthesis in rat liver by 3-month feeding with four kinds of meat protein diets. Fish and pork protein diets upregulated the gene expression involved in cholesterol synthesis and esterification, and pork protein diet also upregulated the gene expression of high-density lipoprotein receptor and low-density lipoprotein receptor. Chicken, pork, and beef protein diets upregulated the gene expression involved in cholesterol reverse transport and bile acid production, which increased total cholesterol level in the fish protein diet group. The total cholesterol levels in liver were lower in the pork and beef protein diet groups. The triglyceride levels in liver were lower in the chicken, pork, and beef protein diet groups. PGC1-α was upregulated by the chicken, pork, and beef protein diets, and promoted the degradation and metabolism of triglyceride, resulting in lower triglyceride in the three diet groups. Meat proteins at the recommended level could be more conducive to cholesterol degradation, triglyceride decomposition, and energy synthesis maintenance at a healthy level. The findings give a new insight into the associations between meat diet and human health.

## Data Availability Statement

The datasets presented in this study can be found in online repositories. The names of the repository/repositories and accession number(s) can be found at: ProteomeXchange with identifier PXD026732.

## Ethics Statement

The animal study was reviewed and approved by the Ethical Committee of Experimental Animal Center of Nanjing Agricultural University.

## Author Contributions

CL and GZ designed the research and provided the funding. XS performed the study and data analysis. ZH performed the study and wrote the manuscript. All authors contributed to the article and approved the submitted version.

## Conflict of Interest

The authors declare that the research was conducted in the absence of any commercial or financial relationships that could be construed as a potential conflict of interest.

## Publisher's Note

All claims expressed in this article are solely those of the authors and do not necessarily represent those of their affiliated organizations, or those of the publisher, the editors and the reviewers. Any product that may be evaluated in this article, or claim that may be made by its manufacturer, is not guaranteed or endorsed by the publisher.

## Abbreviations

*Abca1*: ATP binding cassette subfamily A member 1
*Acat2*: acetyl-Coenzyme A acetyltransferase 2
ADG: average daily weight gain
BCA: bicinchoninic acid assay
CCDC58: coiled-coil domain-containing 58
CISD1: CDGSH iron sulfur domain-containing protein
*Cox*: cytochrome c oxidase subunit
*Cyp7a1*: cytochrome P450 family 7 subfamily A member 1
DTT: dithiothreitol
ENPP1: ectonucleotide pyrophosphatase/phosphodiesterase 1
*Gapdh*: glyceraldehyde-3-phosphate dehydrogenase
GO: gene ontology
GR: glucocorticoid receptor
HCD: high collision dissociation
*Hmgcr*: 3-hydroxy-3-methylglutaryl-coenzyme A reductase
HPR-PF: high pH reversed phase fractionation
IAM: iodoacetamide
IgG: immunoglobulin G
ISCA: iron sulfur cluster-related proteins
ITRAQ: isobaric tag for relative and absolute quantitation
KEGG: Kyoto Encyclopedia of Genes and Genomes
*Lcat*: lecithin-cholesterol acyltransferase
LC–ESI–MS/MS: liquid chromatography electrospray ionization tandem mass spectrometry/mass spectrometry
*Ldlr*: low-density lipoprotein receptor
*mt-Cytb*: mitochondrially encoded cytochrome B
*mt-Nd5*: mitochondrially encoded educed form of nicotinamide-adenine dinucleotid dehydrogenase 5
NADK: NAD kinase
NAUFB: NADH:ubiquinone oxidoreductase subunit B
NCBI: National Center for Biotechnology Information
*Ppargc1a*: peroxisomal proliferator-activated receptor-gamma coactivator-1 alpha
*Ppar*: peroxisome proliferator-activated receptor
PPI: protein-protein interaction
*Rn18s*: 18 S ribosomal RNA
RT-PCR: reverse transcription polymerase chain reaction
SDS-PAGE: sodium dodecyl sulfate polyacrylamide gel electrophoresis
*Scarb1*: scavenger receptor class B type I
*Srebf*: sterol regulatory element-binding transcription factor
TBST: tris-buffered saline and Tween 20
TCA: trichloroacetic acid
TIMM: translocase of inner mitochondrial membrane
TRAP1: TNF receptor-associated protein 1
*Ucp*: uncoupling protein.
